# Synthesis, crystal structure, stereochemical and Hirshfeld surface analysis of *trans*-di­aqua­bis­(1-phenyl­propane-1,2-di­amine-κ^2^ *N*,*N*′)nickel(II) dichloride dihydrate

**DOI:** 10.1107/S2056989023008538

**Published:** 2023-09-29

**Authors:** Shanmugasundaram Akila, Thankakan Vidhyasagar, Aravazhi Amalan Thiruvalluvar, Krishnan Rajeswari

**Affiliations:** a Department of Chemistry, Annamalai University, Annamalai Nagar 608 002, Tamil Nadu, India; b Principal (Retired), Kunthavai Naacchiyaar Government Arts College for Women (Autonomous), Thanjavur 613 007, Tamil Nadu, India; c PG & Research Department of Chemistry, Government Arts College, Chidambaram 608 102, Tamil Nadu, India; Vienna University of Technology, Austria

**Keywords:** synthesis, X-ray crystal structure, Ni^II^ complex, ethyl­enedi­amine metal complex, vicinal di­amine derivative, DFT, Hirshfeld surface analysis, distorted octa­hedral complex, *trans*-Ni complex.

## Abstract

In the hydrated complex salt, [Ni(C_9_H_14_N_2_)_2_(H_2_O)_2_]Cl_2_·2H_2_O, the asymmetric unit comprises of half of the complex cation along with one chloride anion and one non-coordinating water mol­ecule. The central nickel(II) atom is located on an inversion center and is coordinated in a *trans* octa­hedral fashion by four N atoms from two bidentate 1,2-di­amino-1-phenyl­propane ligands in the equatorial plane, and by two oxygen atoms from two water mol­ecules occupying the axial sites. The five-membered chelate ring is in a slightly twisted envelope conformation. The crystal packing features O—H⋯Cl, N—H⋯O and N—H⋯Cl hydrogen bonds. Hirshfeld surface analysis revealed that the most important contributions to the crystal packing are from H⋯H (56.4%), O⋯H/H⋯O (16.4%) and H⋯Cl (13.3%) inter­actions. The crystal void volume was calculated to be 15.17%.

## Abstract 2

The cation of the title compound exhibits point group symmetry 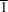 , with the central Ni^II^ atom in a *trans* [N_4_O_2_] coordination environment.

## 1. Chemical context

Unsymmetrically substituted vicinal di­amines are an important class of organic compounds widely used as chelating agents. They are important structural units that have been used for decades, including in asymmetric synthesis. Besides possessing anti­cancer activities (Gayathri *et al.*, 2017 ▸), their metal complexes, being analogues of *cis*-platin, play crucial roles in biological processes including metal-ion-involved metabolism. In addition, such metal complexes find extensive applications in the materials field (Hussain *et al.*, 2019 ▸; Rajeshwari *et al.*, 2021 ▸). Such complexes with nickel(II) and bio-active unsymmetrically substituted vicinal di­amines are inter­esting since nickel is found to be a major trace element, playing a crucial role as a catalytic center in many important metabolic enzymes. Exploring the mol­ecular structure of such Ni^II^ complexes with bio-active di­amine ligands becomes inevitable in order to understand their properties and find possible applications in materials and medicinal chemistry.

In this context, we report here on the synthesis, crystal structure and Hirshfeld surface analysis of the complex salt *trans*-di­aqua­bis­(1-phenyl­propane-1,2-di­amine-κ^2^ *N*,*N*′)nickel(II) dichloride dihydrate, [Ni(C_9_H_14_N_2_)_2_(H_2_O)_2_]Cl_2_·2H_2_O, (I). 
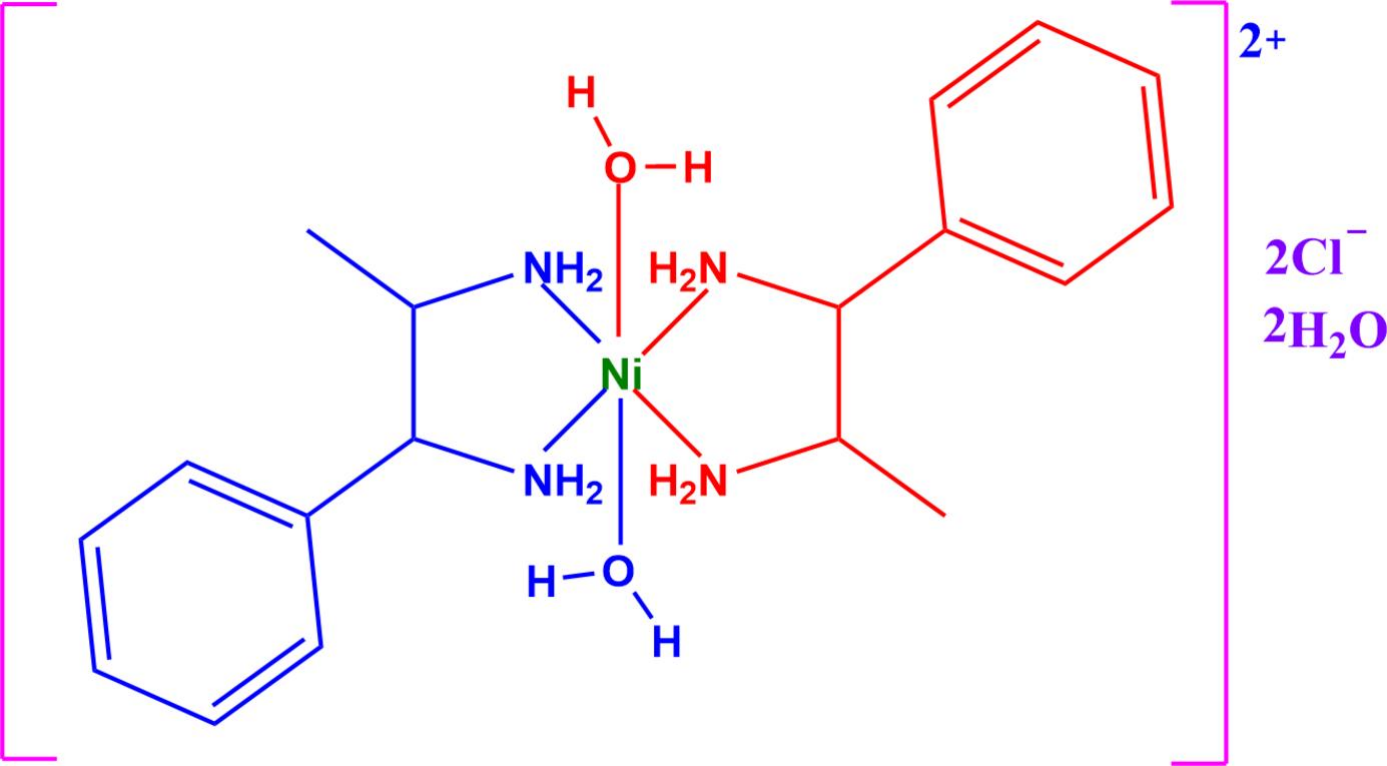


## 2. Structural commentary

**Figure 1:**
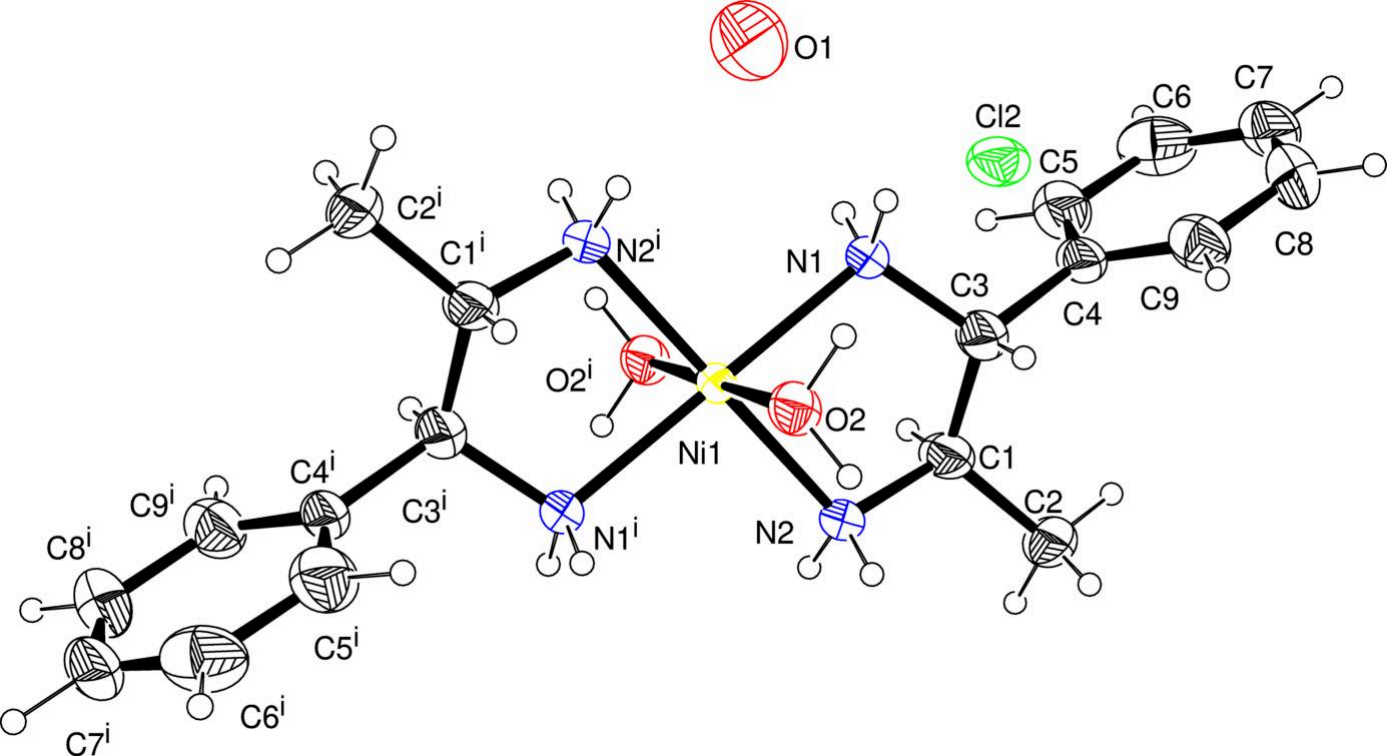
View of the mol­ecular structure of (I), showing displacement ellipsoids at the 30% probability level and spheres of arbitrary radius for the H atoms. [Symmetry code: (i) −*x*, −*y* + 1, −*z* + 1.]

The asymmetric unit of (I) consists of half of the cationic complex, one chloride anion and one non-coordinating water mol­ecule. The central Ni^II^ atom is located on an inversion center, and the other half of the cationic complex is generated by symmetry operation −*x*, −*y* + 1, −*z* + 1. The nickel(II) atom shows a distorted *trans*-octa­hedral coordination by four N atoms from two bidentate 1,2-di­amino-1-phenyl­propane ligands in the equatorial plane, and by two oxygen atoms from two water mol­ecules in the axial sites (Fig. 1 ▸). The five-membered (Ni1/N1/C3/C1/N2) chelate ring is in a slightly twisted envelope conformation with puckering parameters (Cremer & Pople, 1975 ▸) of *Q*(2) = 0.346 (4) Å, φ(2) = 86.6 (4)°, closest pucker descriptor: twisted on C3—C1. The Ni1—O2 bond length is 2.158 (2) Å whereas the Ni1—N bonds are shorter with 2.092 (2) Å to N1 and 2.070 (3) Å to N2. The differences in Ni—N bond lengths may be due to the influence of unsymmetrical substitutions at C3 and C1. The *cis*-N1—Ni1—N2 bond angle is found to be 82.45 (9)° and that of *cis*-N1—Ni1—N2^i^ is 97.55 (9)°. The N1—Ni1—O2 bond angle is 91.79 (9)° and that of N1^i^—Ni1—O2 is 88.21 (9)°. The bond lengths and angles in the complex cation of (I) are comparable with those in similar structures (Sbai *et al.*, 2002 ▸; Li *et al.*, 2005 ▸; Chen *et al.*, 2006 ▸; Kim & Lee, 2002 ▸).

## 3. Supra­molecular features

**Figure 2:**
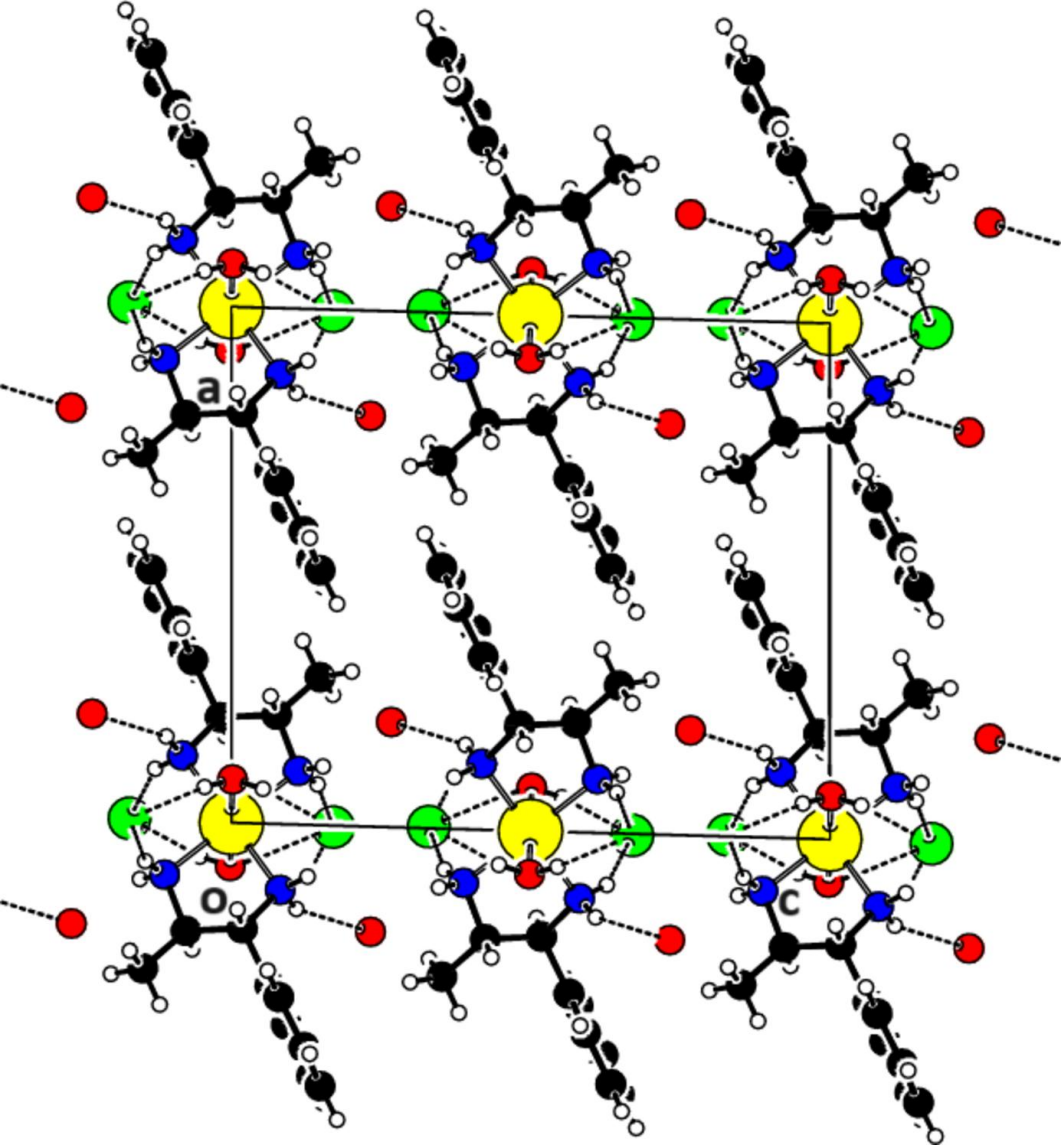
A partial packing diagram of (I) viewed along the *b* axis showing the O—H⋯Cl, N—H⋯O and N—H⋯Cl hydrogen-bonding inter­actions as dashed lines.

The crystal packing of (I) involves hydrogen bonding of the coordinating water mol­ecule (O2) to the chloride anion, and of the amino groups to the non-coordinating water mol­ecule (O1) and the chloride anion (Table 1 ▸, Fig. 2 ▸), establishing a layered arrangement parallel to (100).

## 4. Hirshfeld surface analysis

**Figure 3:**
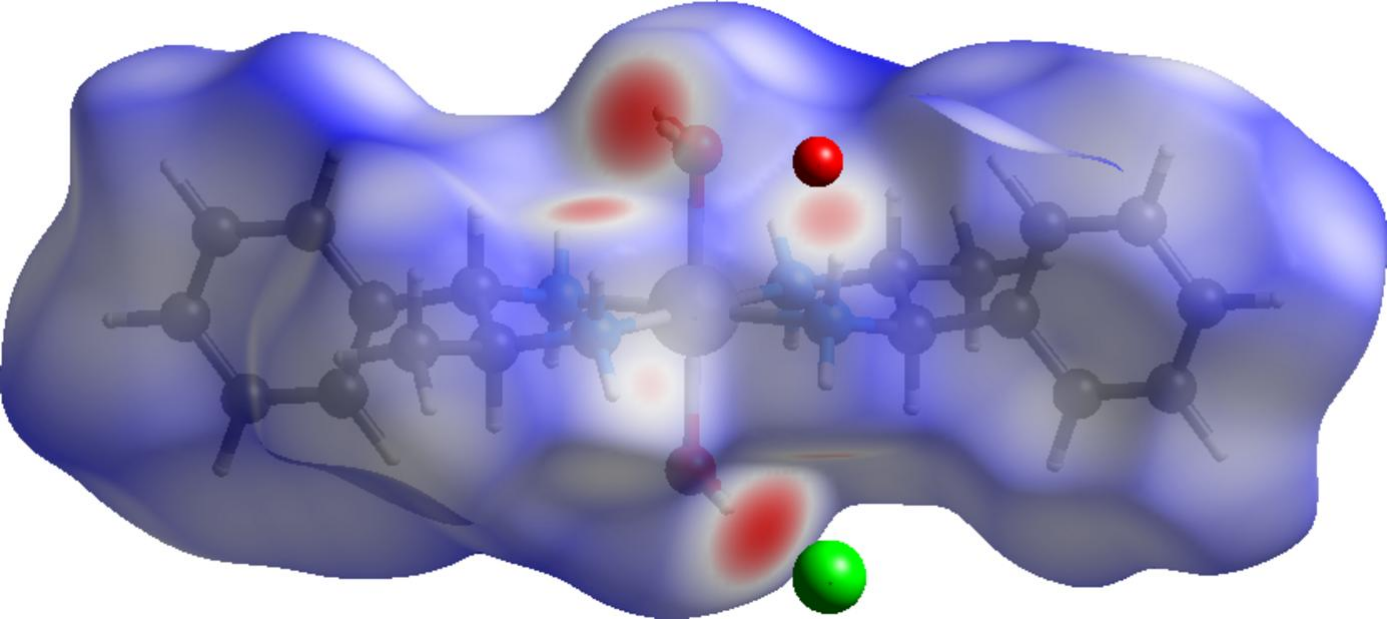
Hirshfeld surface plotted over *d*_norm_ for (I).

**Figure 4:**
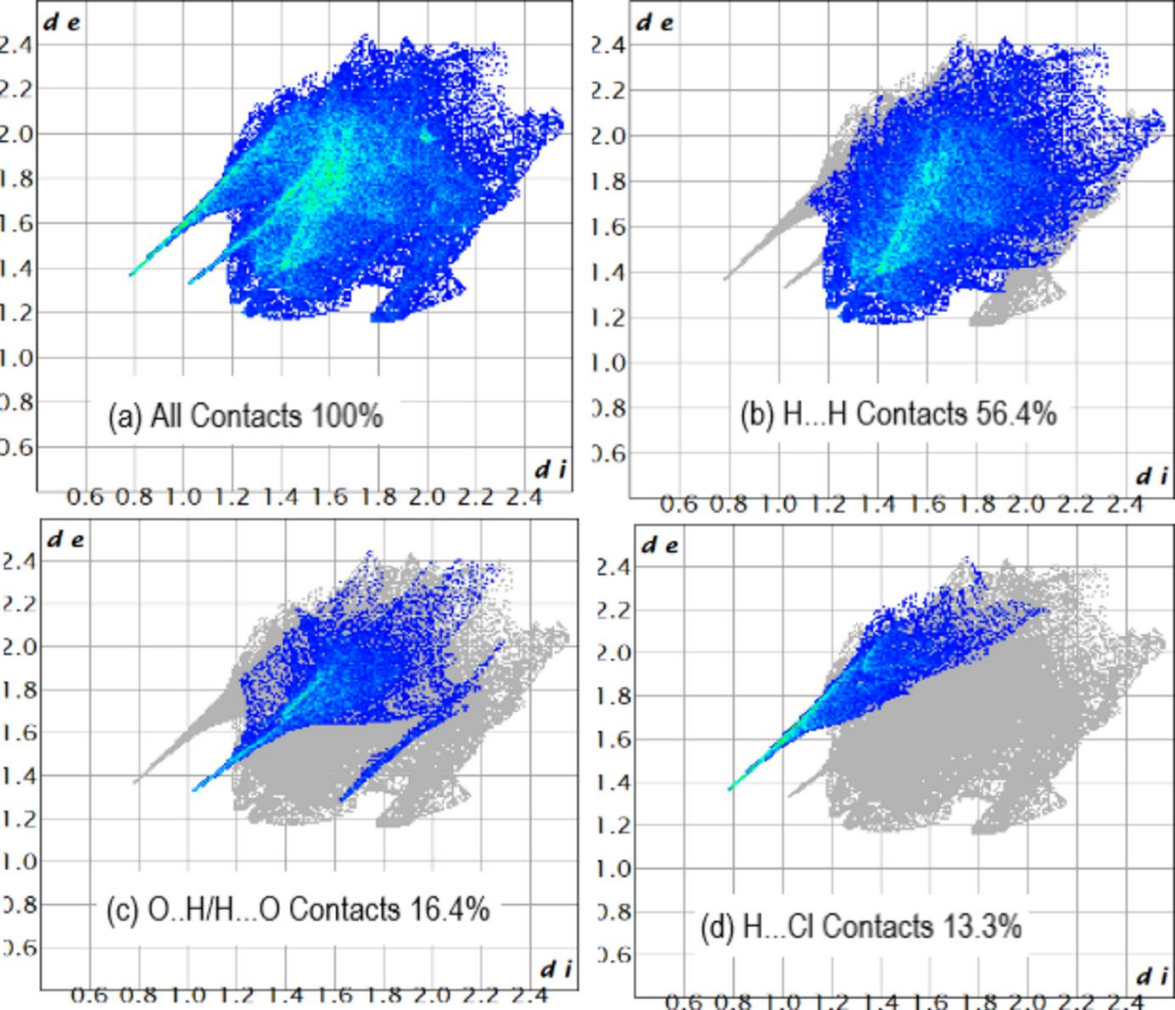
Two-dimensional-fingerprint plots of (I).

The Hirshfeld surface analysis of (I) was carried out with *CrystalExplorer* (Spackman *et al.*, 2021 ▸). Fig. 3 ▸ shows the Hirshfeld surface plotted over *d*_norm_ in the range −0.495 to 1.462 a.u. where the intense red spots represent the shortest inter­molecular contacts between nearest mol­ecules, the blue spots the longest contacts and the white ones medium contacts. In this respect, inter­actions shorter than the van der Waals radii between O—H⋯Cl, N—H⋯Cl or N—H⋯O are shown as bright-red spots. The two-dimensional fingerprint plots are plotted in Fig. 4 ▸. The most important contributions to the crystal packing are from H⋯H (56.4%), O⋯H/H⋯O (16.4%) and H⋯Cl (13.3%) inter­actions.

## 5. HOMO-LUMO

**Figure 5:**
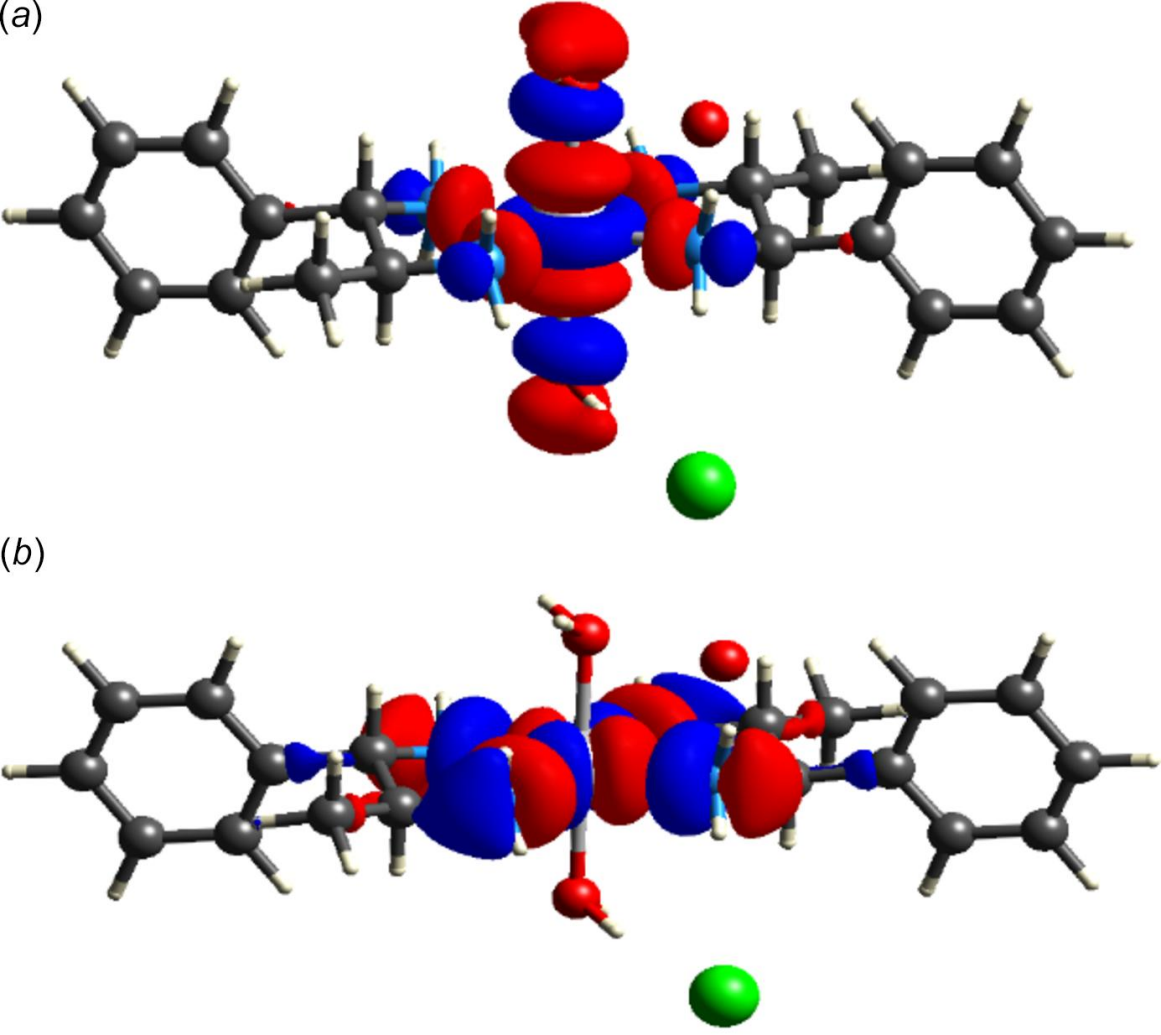
HOMO (*a*) and LUMO (*b*) of (I).

Fig. 5 ▸ represents the HOMO and LUMO of the cationic complex of (I), visualized using *TONTO* calculations in *CrystalExplorer* at the B3LYP/6-31 G(d,p) level. From the HOMO representation, it can be seen that the electrons reside mostly over the metal and water mol­ecules, whereas in the LUMO representation, the electrons are delocalized and largely reside over the metal and amino groups.

## 6. Crystal void

**Figure 6:**
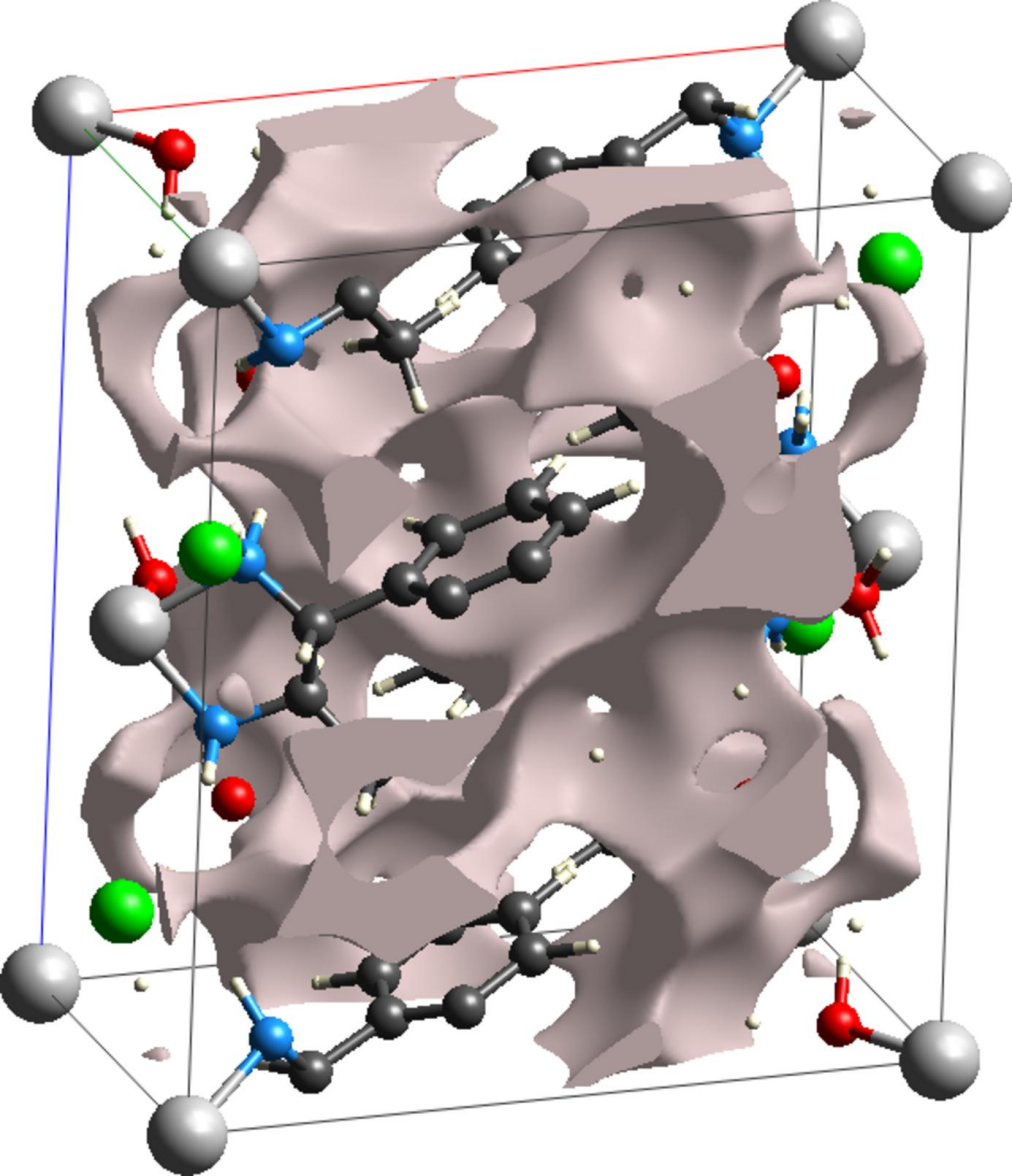
Representation of the crystal voids in the crystal structure of (I).

In order to assess the mechanical stability of the crystal of (I), void analysis (Turner *et al.*, 2011 ▸) was performed with *CrystalExplorer*. The void volume of the crystal of (I) (Fig. 6 ▸), was calculated to be 188.48 Å^3^, *i.e.*, 15.17% of the crystal volume, which shows that the crystal is tightly packed.

## 7. Database survey

**Figure 7:**
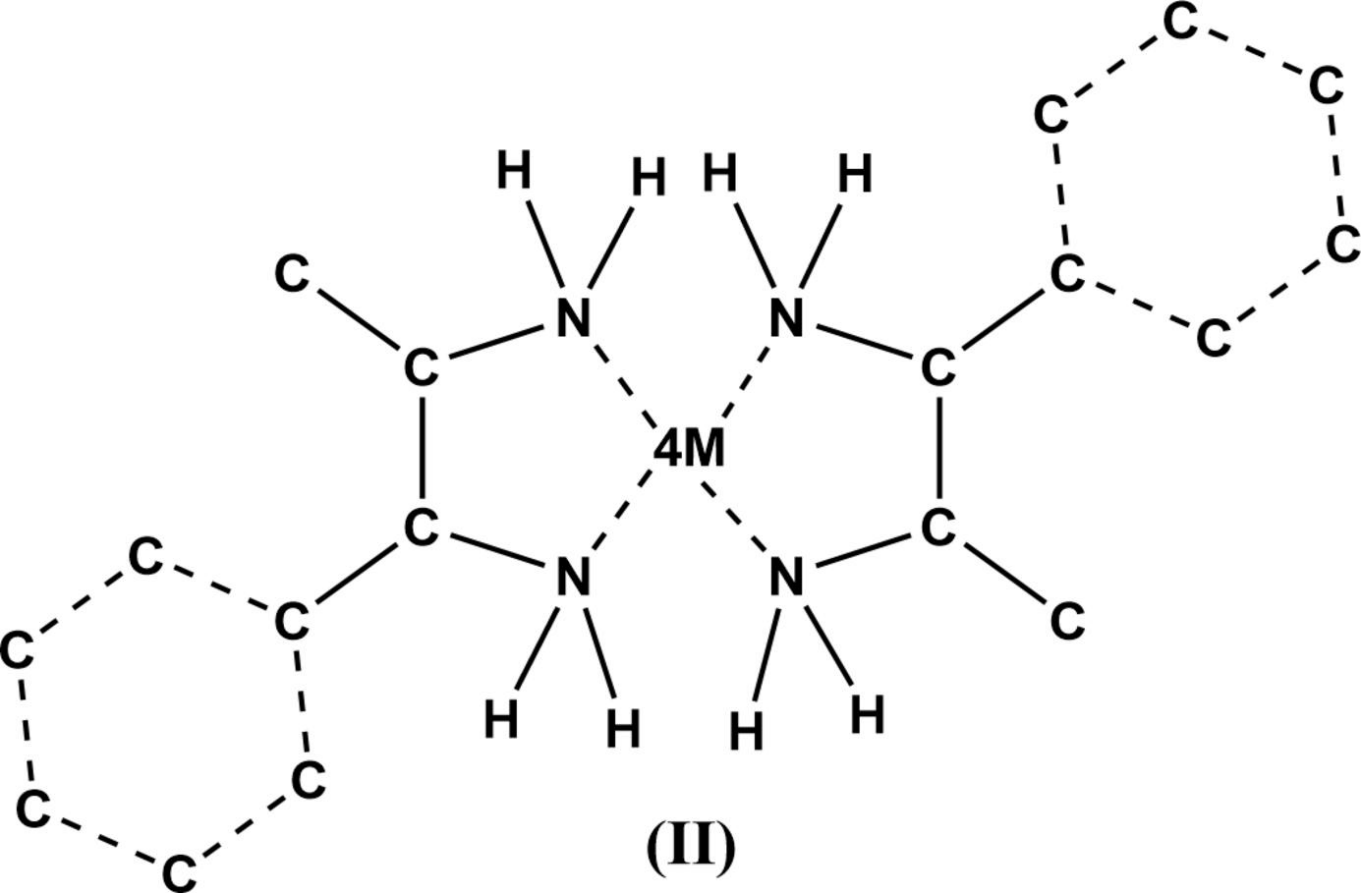
The mol­ecular moiety (II) used for the CSD database search.

A search of the Cambridge Structural Database (CSD, Version 5.44, updated June 2023; Groom *et al.*, 2016 ▸) using the mol­ecular moiety (II) depicted in Fig. 7 ▸ for the basic skeleton of (I), with a transition-metal atom of period 4 at the center, omitting aromatic H, methyl and methine H atoms, water mol­ecules, O and Cl atoms, gave 46 hits. There are no close matches in the CSD since the title compound possesses an unsymmetrically substituted vicinal di­amine ligand.

## 8. Synthesis and crystallization

**Figure 8:**
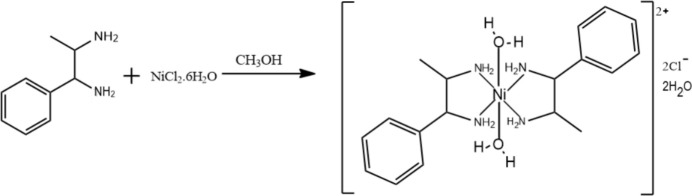
Synthesis scheme of (I).

The salt (I) was synthesized by addition of 1,2-di­amino-1-phenyl­propane (0.02 mol), prepared by the procedure reported by Noller & Baliah (1948 ▸) and Thennarasu & Perumal (2002 ▸), to a nickel dichloride hexa­hydrate (0.01 mol) solution in methanol (20 ml) with stirring under ice-cold conditions. The mixture was stirred in an ice bath for nearly 1 h and the pinkish-red solid formed was filtered and washed with chloro­form. The schematic synthesis is shown in Fig. 8 ▸. Purification and growth of single crystals suitable for X-ray analysis was accomplished by recrystallization from methanol and slow evaporation (m.p. 497 K).

## 9. Refinement

Crystal data, data collection and structure refinement details are summarized in Table 2 ▸. The H atoms attached to C atoms were placed in calculated positions (with aromatic C—H = 0.93, methyl group C—H = 0.96 and methine C—H = 0.98 Å). The H atoms attached to N1 were placed at N1—H1*A* = 0.89 and N1—H1*B* = 0.89 Å. The H atoms attached to N2 were freely refined with N2—H2*C* = 0.84 (5) and N2—H2*D* = 0.81 (5) Å. O1 is the O atom of the non-coordinating water mol­ecule. The two H-atom positions around this O atom were not discernible from difference-Fourier maps and are not included in the model. The H atoms attached to O2 were placed at O2—H2*A* = 0.85 and O2—H2*B* = 0.88 Å. All H atoms (except the freely refined H2*C* and H2*D* atoms) were included as riding contributions with isotropic displacement parameters *U*_iso_(H) = 1.2 and 1.5*U*_eq_(C), 1.2*U*_eq_(N) and 1.5*U*_eq_(O).

## Supplementary Material

Crystal structure: contains datablock(s) I. DOI: 10.1107/S2056989023008538/wm5696sup1.cif

Structure factors: contains datablock(s) I. DOI: 10.1107/S2056989023008538/wm5696Isup3.hkl

Supporting information file. DOI: 10.1107/S2056989023008538/wm5696Isup4.cdx

CCDC reference: 2210342

Additional supporting information:  crystallographic information; 3D view; checkCIF report

## Figures and Tables

**Table 1:** Hydrogen-bond geometry (Å, °)

*D*—H⋯*A*	*D*—H	H⋯*A*	*D*⋯*A*	*D*—H⋯*A*
O2—H2*A*⋯Cl2^i^	0.85	2.29	3.134 (2)	173
O2—H2*B*⋯Cl2	0.88	2.26	3.132 (2)	170
N1—H1*A*⋯O1	0.89	2.48	3.313 (5)	157
N1—H1*B*⋯Cl2	0.89	2.69	3.464 (3)	146
N2—H2*C*⋯Cl2^i^	0.84 (5)	2.68 (5)	3.460 (3)	155 (4)
N2—H2*D*⋯Cl2^ii^	0.81 (5)	2.86 (5)	3.378 (3)	124 (4)

Symmetry codes: (i) 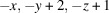 ; (ii) 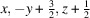 .

**Table 2:** Experimental details

Crystal data	
Chemical formula	[Ni(C_9_H_14_N_2_)_2_(H_2_O)_2_]Cl_2_·2H_2_O
*M*_r_	502.12
Crystal system, space group	Monoclinic, *P*2_1_/*c*
Temperature (K)	298
*a*, *b*, *c* (Å)	12.1130 (7), 7.3062 (4), 14.0441 (8)
β (°)	91.589 (2)
*V* (Å^3^)	1242.42 (12)
*Z*	2
Radiation type	Mo *K*α
μ (mm^−1^)	1.02
Crystal size (mm)	0.31 × 0.27 × 0.21

Data collection	
Diffractometer	Bruker D8 Quest XRD
Absorption correction	Multi-scan (*SADABS*; Krause *et al.*, 2015 ▸)
*T*_min_, *T*_max_	0.634, 0.746
No. of measured, independent and observed [*I* > 2σ(*I*)] reflections	15989, 3619, 2841
*R*_int_	0.020
(sin θ/λ)_max_ (Å^−1^)	0.704

Refinement	
*R*[*F* ^2^ > 2σ(*F* ^2^)], *wR*(*F* ^2^), *S*	0.061, 0.140, 1.08
No. of reflections	3619
No. of parameters	144
H-atom treatment	H atoms treated by a mixture of independent and constrained refinement
Δρ_max_, Δρ_min_ (e Å^−3^)	1.52, −0.91

Computer programs: *APEX3* and *SAINT* (Bruker, 2017 ▸), *SHELXT* (Sheldrick, 2015*a* ▸), *SHELXL* (Sheldrick, 2015*b* ▸), *ORTEP-3 for Windows* (Farrugia, 2012 ▸), *PLATON* (Spek, 2020 ▸) and *publCIF* (Westrip, 2010 ▸).

## supplementary crystallographic information

### Crystal data

**Table:**

[Ni(C_9_H_14_N_2_)_2_(H_2_O)_2_]Cl_2_·2H_2_O	*D*_x_ = 1.331 Mg m^−^^3^
*M**_r_* = 502.12	Melting point: 497 K
Monoclinic, *P*2_1_/*c*	Mo *K*α radiation, λ = 0.71073 Å
*a* = 12.1130 (7) Å	Cell parameters from 7663 reflections
*b* = 7.3062 (4) Å	θ = 3.1–30.0°
*c* = 14.0441 (8) Å	µ = 1.02 mm^−^^1^
β = 91.589 (2)°	*T* = 298 K
*V* = 1242.42 (12) Å^3^	Block, pink
*Z* = 2	0.31 × 0.27 × 0.21 mm
*F*(000) = 524	

### Data collection

**Table:**

Bruker D8 Quest XRD diffractometer	2841 reflections with *I* > 2σ(*I*)
Detector resolution: 7.3910 pixels mm^-1^	*R*_int_ = 0.020
ω and φ scans	θ_max_ = 30.0°, θ_min_ = 3.1°
Absorption correction: multi-scan (*SADABS*; Krause *et al.*, 2015)	*h* = −17→16
*T*_min_ = 0.634, *T*_max_ = 0.746	*k* = −10→10
15989 measured reflections	*l* = −19→17
3619 independent reflections	

### Refinement

**Table:**

Refinement on *F*^2^	Secondary atom site location: difference Fourier map
Least-squares matrix: full	Hydrogen site location: mixed
*R*[*F*^2^ > 2σ(*F*^2^)] = 0.061	H atoms treated by a mixture of independent and constrained refinement
*wR*(*F*^2^) = 0.140	*w* = 1/[σ^2^(*F*_o_^2^) + (0.0335*P*)^2^ + 2.3825*P*] where *P* = (*F*_o_^2^ + 2*F*_c_^2^)/3
*S* = 1.08	(Δ/σ)_max_ < 0.001
3619 reflections	Δρ_max_ = 1.52 e Å^−^^3^
144 parameters	Δρ_min_ = −0.91 e Å^−^^3^
0 restraints	Extinction correction: SHELXL-2019/3 (Sheldrick, 2015*b*), Fc^*^=kFc[1+0.001xFc^2^λ^3^/sin(2θ)]^-1/4^
Primary atom site location: structure-invariant direct methods	Extinction coefficient: 0.011 (2)

### Special details

**Table:**

Geometry. All esds (except the esd in the dihedral angle between two l.s. planes) are estimated using the full covariance matrix. The cell esds are taken into account individually in the estimation of esds in distances, angles and torsion angles; correlations between esds in cell parameters are only used when they are defined by crystal symmetry. An approximate (isotropic) treatment of cell esds is used for estimating esds involving l.s. planes.

### Fractional atomic coordinates and isotropic or equivalent isotropic displacement parameters (Å^2^)

**Table:**

	*x*	*y*	*z*	*U*_iso_*/*U*_eq_	
Ni1	0.000000	0.500000	0.500000	0.03444 (17)	
O1	0.2039 (4)	0.2818 (7)	0.2674 (3)	0.1230 (15)	
O2	−0.08403 (19)	0.7604 (3)	0.49802 (16)	0.0480 (5)	
H2A	−0.067927	0.830472	0.544916	0.072*	
H2B	−0.067224	0.824263	0.447313	0.072*	
Cl2	0.00298 (9)	0.99682 (12)	0.32983 (6)	0.0590 (3)	
N1	0.1278 (2)	0.6032 (3)	0.41843 (16)	0.0393 (5)	
H1A	0.163790	0.511552	0.391321	0.047*	
H1B	0.100305	0.675472	0.372616	0.047*	
N2	0.1041 (2)	0.5800 (4)	0.61171 (18)	0.0402 (5)	
C1	0.2120 (3)	0.6298 (7)	0.5760 (2)	0.0656 (11)	
H1	0.247413	0.511203	0.565627	0.079*	
C2	0.2870 (3)	0.7202 (6)	0.6481 (3)	0.0608 (10)	
H2E	0.356214	0.748091	0.619709	0.091*	
H2F	0.299490	0.639331	0.701219	0.091*	
H2G	0.253580	0.831281	0.669762	0.091*	
C3	0.2038 (3)	0.7078 (7)	0.4816 (3)	0.0670 (11)	
H3	0.167920	0.826718	0.489949	0.080*	
C4	0.3132 (3)	0.7513 (6)	0.4357 (2)	0.0568 (9)	
C5	0.3882 (4)	0.6251 (7)	0.4081 (3)	0.0793 (13)	
H5	0.374550	0.501135	0.416994	0.095*	
C6	0.4867 (4)	0.6819 (9)	0.3659 (4)	0.0890 (16)	
H6	0.538556	0.595730	0.347740	0.107*	
C7	0.5056 (3)	0.8635 (9)	0.3517 (3)	0.0806 (15)	
H7	0.570103	0.901948	0.323347	0.097*	
C8	0.4309 (4)	0.9858 (7)	0.3788 (4)	0.0824 (14)	
H8	0.443916	1.109789	0.368966	0.099*	
C9	0.3354 (3)	0.9320 (7)	0.4208 (3)	0.0669 (10)	
H9	0.284926	1.020016	0.439403	0.080*	
H2C	0.079 (3)	0.667 (6)	0.644 (3)	0.068 (13)*	
H2D	0.114 (4)	0.503 (6)	0.653 (3)	0.076 (14)*	

### Atomic displacement parameters (Å^2^)

**Table:**

	*U*^11^	*U*^22^	*U*^33^	*U*^12^	*U*^13^	*U*^23^
Ni1	0.0403 (3)	0.0322 (3)	0.0311 (3)	−0.0052 (2)	0.00578 (18)	0.00010 (19)
O1	0.126 (3)	0.115 (3)	0.128 (4)	0.001 (3)	0.006 (3)	0.009 (3)
O2	0.0588 (13)	0.0360 (11)	0.0495 (12)	0.0009 (10)	0.0076 (10)	0.0012 (9)
Cl2	0.0953 (7)	0.0415 (4)	0.0409 (4)	0.0030 (4)	0.0133 (4)	−0.0025 (3)
N1	0.0426 (12)	0.0424 (13)	0.0331 (11)	−0.0084 (10)	0.0038 (9)	0.0001 (10)
N2	0.0450 (13)	0.0439 (14)	0.0318 (12)	−0.0060 (11)	0.0047 (10)	0.0005 (11)
C1	0.0497 (19)	0.107 (3)	0.0399 (17)	−0.026 (2)	0.0018 (14)	0.0001 (19)
C2	0.0518 (19)	0.082 (3)	0.0479 (19)	−0.0132 (19)	−0.0065 (15)	−0.0045 (18)
C3	0.058 (2)	0.095 (3)	0.0470 (19)	−0.031 (2)	0.0043 (16)	−0.0007 (19)
C4	0.0435 (17)	0.086 (3)	0.0412 (16)	−0.0226 (18)	0.0046 (13)	−0.0004 (17)
C5	0.102 (4)	0.063 (3)	0.074 (3)	−0.008 (3)	0.015 (3)	0.002 (2)
C6	0.072 (3)	0.120 (5)	0.076 (3)	0.029 (3)	0.012 (2)	−0.008 (3)
C7	0.049 (2)	0.126 (5)	0.068 (3)	−0.027 (3)	0.0143 (18)	0.000 (3)
C8	0.084 (3)	0.079 (3)	0.085 (3)	−0.027 (3)	0.019 (3)	0.007 (3)
C9	0.057 (2)	0.080 (3)	0.065 (2)	−0.001 (2)	0.0106 (18)	0.005 (2)

### Geometric parameters (Å, º)

**Table:**

Ni1—N2^i^	2.070 (3)	C2—H2E	0.9600
Ni1—N2	2.070 (3)	C2—H2F	0.9600
Ni1—N1^i^	2.092 (2)	C2—H2G	0.9600
Ni1—N1	2.092 (2)	C3—C4	1.523 (5)
Ni1—O2^i^	2.158 (2)	C3—H3	0.9800
Ni1—O2	2.158 (2)	C4—C5	1.359 (6)
O2—H2A	0.8524	C4—C9	1.364 (6)
O2—H2B	0.8799	C5—C6	1.409 (7)
N1—C3	1.475 (4)	C5—H5	0.9300
N1—H1A	0.8900	C6—C7	1.362 (8)
N1—H1B	0.8900	C6—H6	0.9300
N2—C1	1.458 (4)	C7—C8	1.335 (7)
N2—H2C	0.84 (5)	C7—H7	0.9300
N2—H2D	0.81 (5)	C8—C9	1.371 (6)
C1—C3	1.444 (5)	C8—H8	0.9300
C1—C2	1.496 (5)	C9—H9	0.9300
C1—H1	0.9800		
			
N2^i^—Ni1—N2	180.0	C3—C1—H1	103.4
N2^i^—Ni1—N1^i^	82.45 (9)	N2—C1—H1	103.4
N2—Ni1—N1^i^	97.55 (9)	C2—C1—H1	103.4
N2^i^—Ni1—N1	97.55 (9)	C1—C2—H2E	109.5
N2—Ni1—N1	82.45 (9)	C1—C2—H2F	109.5
N1^i^—Ni1—N1	180.0	H2E—C2—H2F	109.5
N2^i^—Ni1—O2^i^	92.17 (11)	C1—C2—H2G	109.5
N2—Ni1—O2^i^	87.83 (11)	H2E—C2—H2G	109.5
N1^i^—Ni1—O2^i^	91.79 (9)	H2F—C2—H2G	109.5
N1—Ni1—O2^i^	88.21 (9)	C1—C3—N1	111.9 (3)
N2^i^—Ni1—O2	87.83 (11)	C1—C3—C4	115.7 (3)
N2—Ni1—O2	92.17 (11)	N1—C3—C4	112.9 (3)
N1^i^—Ni1—O2	88.21 (9)	C1—C3—H3	105.0
N1—Ni1—O2	91.79 (9)	N1—C3—H3	105.0
O2^i^—Ni1—O2	180.00 (11)	C4—C3—H3	105.0
Ni1—O2—H2A	114.9	C5—C4—C9	118.5 (4)
Ni1—O2—H2B	111.0	C5—C4—C3	125.1 (4)
H2A—O2—H2B	104.7	C9—C4—C3	116.4 (4)
C3—N1—Ni1	108.45 (19)	C4—C5—C6	120.1 (5)
C3—N1—H1A	110.0	C4—C5—H5	120.0
Ni1—N1—H1A	110.0	C6—C5—H5	120.0
C3—N1—H1B	110.0	C7—C6—C5	119.8 (5)
Ni1—N1—H1B	110.0	C7—C6—H6	120.1
H1A—N1—H1B	108.4	C5—C6—H6	120.1
C1—N2—Ni1	110.06 (19)	C8—C7—C6	119.5 (4)
C1—N2—H2C	110 (3)	C8—C7—H7	120.2
Ni1—N2—H2C	114 (3)	C6—C7—H7	120.2
C1—N2—H2D	107 (3)	C7—C8—C9	121.1 (5)
Ni1—N2—H2D	115 (3)	C7—C8—H8	119.4
H2C—N2—H2D	101 (4)	C9—C8—H8	119.4
C3—C1—N2	112.0 (3)	C4—C9—C8	121.1 (4)
C3—C1—C2	118.2 (4)	C4—C9—H9	119.5
N2—C1—C2	114.3 (3)	C8—C9—H9	119.5
			
Ni1—N2—C1—C3	32.1 (5)	C1—C3—C4—C9	−113.5 (5)
Ni1—N2—C1—C2	169.9 (3)	N1—C3—C4—C9	115.7 (4)
N2—C1—C3—N1	−44.5 (5)	C9—C4—C5—C6	0.6 (7)
C2—C1—C3—N1	179.4 (4)	C3—C4—C5—C6	179.8 (4)
N2—C1—C3—C4	−175.8 (4)	C4—C5—C6—C7	−0.9 (8)
C2—C1—C3—C4	48.2 (6)	C5—C6—C7—C8	0.6 (8)
Ni1—N1—C3—C1	33.9 (4)	C6—C7—C8—C9	0.1 (8)
Ni1—N1—C3—C4	166.5 (3)	C5—C4—C9—C8	0.1 (7)
C1—C3—C4—C5	67.3 (6)	C3—C4—C9—C8	−179.2 (4)
N1—C3—C4—C5	−63.4 (5)	C7—C8—C9—C4	−0.4 (7)

Symmetry code: (i) −*x*, −*y*+1, −*z*+1.

### Hydrogen-bond geometry (Å, º)

**Table:**

*D*—H···*A*	*D*—H	H···*A*	*D*···*A*	*D*—H···*A*
O2—H2*A*···Cl2^ii^	0.85	2.29	3.134 (2)	173
O2—H2*B*···Cl2	0.88	2.26	3.132 (2)	170
N1—H1*A*···O1	0.89	2.48	3.313 (5)	157
N1—H1*B*···Cl2	0.89	2.69	3.464 (3)	146
N2—H2*C*···Cl2^ii^	0.84 (5)	2.68 (5)	3.460 (3)	155 (4)
N2—H2*D*···Cl2^iii^	0.81 (5)	2.86 (5)	3.378 (3)	124 (4)

Symmetry codes: (ii) −*x*, −*y*+2, −*z*+1; (iii) *x*, −*y*+3/2, *z*+1/2.

## References

[bb1] Bruker (2017). *APEX3* and *SAINT*. Bruker AXS Inc., Madison, Wisconsin, USA.

[bb2] Chen, Z.-L., Zhang, Y.-Z. & Liang, F.-P. (2006). *Acta Cryst.* E**62**, m2287–m2289.

[bb3] Cremer, D. & Pople, J. A. (1975). *J. Am. Chem. Soc.* **97**, 1354–1358.

[bb4] Farrugia, L. J. (2012). *J. Appl. Cryst.* **45**, 849–854.

[bb5] Gayathri, A., Rajeswari, K., Vidhyasagar, T. & Selvanayagam, S. (2017). *Acta Cryst.* E**73**, 1878–1881. 10.1107/S2056989017016292 PMC5730244 29250407

[bb6] Groom, C. R., Bruno, I. J., Lightfoot, M. P. & Ward, S. C. (2016). *Acta Cryst.* B**72**, 171–179. 10.1107/S2052520616003954 PMC4822653 27048719

[bb7] Hussain, A., AlAjmi, M. F., Rehman, Md. T., Amir, S., Husain, F. M., Alsalme, A., Siddiqui, M. A., Alkhedairy, A. A. & Khan, R. A. (2019). *Sci. Rep.* **9**, 5237, 1–17. 10.1038/s41598-019-41063-x PMC6437194 30918270

[bb8] Kim, C.-H. & Lee, S.-G. (2002). *Acta Cryst.* C**58**, m421–m423. 10.1107/s0108270102009897 12094047

[bb9] Krause, L., Herbst-Irmer, R., Sheldrick, G. M. & Stalke, D. (2015). *J. Appl. Cryst.* **48**, 3–10. 10.1107/S1600576714022985 PMC4453166 26089746

[bb10] Li, M. T., Wang, C.-G., Wu, Y. & Fu, X.-C. (2005). *Acta Cryst.* E**61**, m1613–m1615.

[bb11] Noller, C. R. & Baliah, V. (1948). *J. Am. Chem. Soc.* **70**, 3853–3855. 10.1021/ja01191a092 18121891

[bb12] Rajeshwari, K., Anantha Lakshmi, P. V., Archana, J. & Sumakanth, M. (2021). *Appl. Organom Chem.* **35**, e6100.

[bb13] Sbai, F., Chkirate, K., Regragui, R., Essassi, E. M. & Pierrot, M. (2002). *Acta Cryst.* E**58**, m337–m339. 10.1107/s0108270103013945 12909760

[bb14] Sheldrick, G. M. (2015*a*). *Acta Cryst.* A**71**, 3–8.

[bb15] Sheldrick, G. M. (2015*b*). *Acta Cryst.* C**71**, 3–8.

[bb16] Spackman, P. R., Turner, M. J., McKinnon, J. J., Wolff, S. K., Grimwood, D. J., Jayatilaka, D. & Spackman, M. A. (2021). *J. Appl. Cryst.* **54**, 1006–1011. 10.1107/S1600576721002910 PMC8202033 34188619

[bb17] Spek, A. L. (2020). *Acta Cryst.* E**76**, 1–11. 10.1107/S2056989019016244 PMC6944088 31921444

[bb18] Thennarasu, S. & Perumal, P. T. (2002). *Molecules*, **7**, 487–493.

[bb19] Turner, M. J., McKinnon, J. J., Jayatilaka, D. & Spackman, M. A. (2011). *CrystEngComm*, **13**, 1804–1813.

[bb20] Westrip, S. P. (2010). *J. Appl. Cryst.* **43**, 920–925.

